# Human DESC1 serine protease confers tumorigenic properties to MDCK cells and it is upregulated in tumours of different origin

**DOI:** 10.1038/sj.bjc.6603856

**Published:** 2007-06-19

**Authors:** C G Viloria, J R Peinado, A Astudillo, O García-Suárez, M V González, C Suárez, S Cal

**Affiliations:** 1 Departamento de Bioquímica y Biología Molecular, Instituto Universitario de Oncología, Universidad de Oviedo, 33006, Asturias, Spain; 2 Departamentos de Anatomia Patologica, Hospital Universitario Central de Asturias, Instituto Universitario de Oncología, Oviedo, 33006, Asturias, Spain; 3 y Otorrinolaringología, Hospital Universitario Central de Asturias, Instituto Universitario de Oncología, Oviedo, 33006, Asturias, Spain

**Keywords:** degradome, pericellular proteolysis, extracellular matrix

## Abstract

Proteolysis of the extracellular matrix components plays a crucial role in the regulation of the cellular and physiological processes, and different pathologies have been associated with the loss or gain of function of proteolytic enzymes. DESC1 (differentially expressed in squamous cell carcinoma gene 1), a member of the TTSP (type II transmembrane serine protease) family of serine proteases, is an epithelial-specific enzyme that has been found downregulated in squamous cell carcinoma of the head and neck region. We describe new properties of DESC1 suggesting that this protease may be involved in the progression of some type of tumours. Thus, this enzyme hydrolyses some extracellular matrix components, such as fibronectin, gelatin or fibrinogen. Moreover, Madin–Darby canine kidney (MDCK) cells expressing exogenous human DESC1 acquire properties associated with tumour growth such as enhanced motility and an increase of tubular forms in a 3D collagen lattice following HGF treatment. Finally, we generated polyclonal anti-DESC1 antibodies and immunohistochemical analysis in tissues different from head and neck region indicated that this protease was overexpressed in tumours of diverse origins. Taken together, our results suggest that DESC1 could be considered as a potential therapeutic target in some type of tumours.

Pericellular proteolysis is a crucial biological event: membrane-associated proteolytic enzymes are involved in dynamic rearrangements in cell–cell and cell–matrix interactions and deregulation of these activities underlies different pathologies, including cancer (Freije *et al*, 2003). In fact, the uncontrolled degradation of the extracellular matrix allows the tumour cells to invade surrounding tissues and spread throughout the body. Thus, it is easy to understand that the identification and further characterisation of the proteolytic systems constitute an important challenge for biomedical research, since it may help to identify accurate molecular markers to detect the metastatic disease (Overall and Lopez-Otin, 2002).

The TTSP (type II transmembrane serine proteases) constitute a family of membrane-anchored serine proteases of growing interest since many members have been found widely deregulated during tumour development and progression (Netzel-Arnett *et al*, 2003). These proteases are structurally complex enzymes containing the catalytic domain at the carboxy terminal region. This domain possesses three highly conserved residues (His, Asp and Ser), which form the catalytic triad of the enzyme. The amino terminal region includes the transmembrane region and an activation domain to switch the protein to its active form. Between these two regions, a variable number of potential protein–protein interaction modular domains can be found. More than 17 members in humans and 20 TTSP genes in mice have been identified at present (Szabo *et al*, 2005). Although the specific role of most of these enzymes remains unresolved, available information indicates that the TTSP are involved in a variety of key biological functions. Thus, matriptase is an essential enzyme for postnatal survival, epidermal barrier function, hair follicle development and thymic homeostasis in mice (List *et al*, 2002, 2006); HAT (human airway trypsin-like protease) has been proposed to play a role in host immune defence (Yamaoka *et al*, 1998); corin regulates blood pressure by activating the cardiac hormone pro-ANP (Yan *et al*, 2000); and TMPRSS3 has been suggested to be involved in inner ear development (Guipponi *et al*, 2002). Likewise, the role of the TTSP in tumour growth, cancer invasion and metastasis processes are being increasingly documented for proteases such as matriptase (List *et al*, 2005), matriptase-2 (Velasco *et al*, 2002) and hepsin (Hobson *et al*, 2004; Klezovitch *et al*, 2004).

The HAT/DESC constitutes a phylogenetically related subfamily of TTSP (Netzel-Arnett *et al*, 2003) with five *DESC1* (differentially expressed in squamous cell carcinoma gene 1)-like genes clustered within a region in the chromosome 4q (Behrens *et al*, 2004; Hobson *et al*, 2004). *DESC1* was identified through the reduced levels of associated mRNA present in tumours from diverse sites in the head and neck region when compared with corresponding normal tissue (Lang and Schuller, 2001). Recently, the protein has been reported to be downregulated in tissues from the oropharyngeal cavity during the squamous cell carcinoma progression and upregulated during normal epithelial differentiation (Sedghizadeh *et al*, 2006).

Our interest in genes differentially expressed in head and neck carcinomas has directed our attention to DESC1. In this report we describe new insights about the biochemical and functional properties of this protease indicating that this enzyme could be involved in tumour progression. Thus, this protease can hydrolyse different extracellular matrix components and also can activate pro-uPA. Likewise, different assays using Madin–Darby canine kidney (MDCK) cells stably transfected with DESC1 full-length cDNA showed that this enzyme enhances cell motility. Moreover, these cells form long extensions when they are grown in 3D collagen lattice, which represents a partial epithelial–mesenchymal transition (O’Brien *et al*, 2002). Finally, the generation of antibodies towards its catalytic domain has allowed us to find that DESC1 is overexpressed in a variety of previously untested tumours of different origins.

## MATERIALS AND METHODS

### Molecular cloning of human DESC1

The *DESC1* cDNA sequence (GenBank accesion number AF064819) was used as query to carry out a search in the NCBI human Expression Sequence Tag (EST) database (www.ncbi.nlm.nih.gov/Blast/Blast.cgi). An EST sequence from a skin cDNA library, BG697702, was identified and purchased from the Geneservice Ltd (Cambridge, UK). This EST served as template for a PCR amplification of the human *DESC1* full-length cDNA using specific primers. The amplification product was cloned into the *Eco*RV site of the mammalian vector pcDNA3 containing a haemagglutinin (HA) epitope at the C terminus to give a pcDNA-HA-*DESC1* vector. The identity of the sequence was confirmed by automated nucleotide sequencing.

### Production and purification of recombinant catalytic domain DESC1, generation of polyclonal antibodies and Western blot analysis

A 695-bp fragment of the *DESC1* cDNA encoding the entire serine protease domain was generated by PCR amplification using the EST BG697702 as template and the specific oligonucleotides *desc3F* (5′-ATCGTTGGTGGGACAGAAGTAG-3′) and *desc1R* (5′-GATACCAGTTTTTGAAGTAATCCAG-3′). PCR amplification conditions, cloning in pGEX-3X vector, and expression and purification of DESC1 catalytic domain fused to GST were carried out as described to characterise matriptase-2 (Velasco *et al*, 2002), with minor modifications. The resulting expression vector (pGEX-3X-DESC1) was transformed into BL21(DE3)pLysS competent *E. coli* cells, and expression was induced by the addition of isopropyl-1-thio-*β*-D-galactopyranoside (final concentration, 0.5 mM) followed by further incubation for 4–6 h at 30°C. The cells were collected by centrifugation, washed and resuspended in 0.05 volumes of PBS. Finally, the cells were lysed by sonication and centrifuged at 20 000 **g** for 20 min at 4°C. The soluble extract was loaded on a glutathione-Sepharose 4B column (GE Healthcare, Uppsala, Sweden), and the glutathione *S*-transferase GST-DESC1 fusion protein eluted with glutathione elution buffer (10-mM reduced glutathione in 50 mM Tris–HCl, pH 8.0), following the manufacturer's instructions. Finally, the purified GST-DESC1 fusion protein was used for enzymatic assays. GST alone was also produced using same experimental procedures to be used as a control in further assays. Recombinant DESC1 catalytic domain was likewise employed to generate polyclonal antibodies against DESC1, following the same strategy used to generate anti-polyserase-1 antibodies (Cal *et al*, 2003). For Western blot analysis, proteins were separated on SDS–PAGE gels, blotted onto nitrocellulose membranes and incubated following standard procedures.

### Gelatin zymography

A 0.5 *μ*g of GST or GST-DESC1 were mixed with SDS sample buffer in absence of reducting agents. Then, and without boiling, the samples were subjected to electrophoresis in a 12% acrylamide gel containing 0.2% gelatine. Gel was run at 10 mA, washed in 2.5% Triton X-100 for 3 h and incubated at 37°C for 16 h in 20 mM Tris–HCl pH 7.4, and 5 mM CaCl_2_. After incubation, the gel was stained with Coomassie Brilliant Blue R-250, and destained in 30% methanol and 10% acetic acid. Gelatinolyc activities were detected as clear bands in the blue background. As positive control 5 *μ*l of HT1080 conditioned medium were added to one of the lanes (Stanton *et al*, 1998).

### Enzymatic assays

To detect the enzymatic activity of the purified enzyme, recombinant DESC1 was incubated with 5 *μ*g of different proteins including type I laminin, type I gelatin, fibronectin and fibrinogen or 1 *μ*g of pro-uPA. Reactions were carried out and visualised as described in Velasco *et al* (2002). For the inhibition assays, recombinant protein was previously incubated for 30 min at 37°C with 20 *μ*M AEBSF, 0.2 mM TPCK, 2.5 mM EDTA or 35 *μ*M E64, and the subsequent reactions were performed using fibrinogen as substrate. Synthetic peptides Boc-Gln-Gly-Arg-AMC and Boc-Gln-Ala-Arg-AMC were likewise used to detect enzymatic activity, and assays were carried out using 10 *μ*M of substrate and 40 ng of DESC1. Inhibition was likewise tested preincubating the enzyme for 30 min at 37°C with 2.5 mM EDTA; 20, 200 and 500 *μ*M AEBSF; 35 *μ*M E-64; 20 nM antitrypsin or 20 nM antichymotrypsin. Initial velocities were calculated using the analysis package FL WinLab 2.01 (PerkinElmer Life Sciences, Waltham, MA, USA), and data were fitted to the Michaelis–Menten equation using GraFit version 4.0 (Erithacus, Surrey, UK). Purified GST was used as a negative control under the same experimental conditions.

### Cell culture conditions, transfection and immunofluorescence

MDCK cell line was routinely maintained in DMEM media supplemented and 10% fetal bovine serum (FBS) (Invitrogen, Karlsruhe, Germany). MDCK clones expressing recombinant DESC1 (MDCK/DESC1 cells) and MDCK transfected with an empty vector (control cells) were obtained by transfection using the LipofectAMINE reagent (Invitrogen). Stable transfection in MDCK cells was carried out in DMEM medium containing 800 *μ*g ml^−1^ of geneticin. To detect recombinant DESC1, cells were fixed with 4% paraformaldehyde in phosphate-buffered saline, and blocked with 15% of FBS. Then, the slides were incubated for 2 h with a primary anti-HA (Roche, Basel, Switzerland) or anti-DESC1 antibody, followed by 2 h of incubation with a secondary fluorescein-conjugated sheep anti-mouse antibody (GE Healthcare) or a Texas Red-conjugated donkey anti-rabbit secondary antibody (GE Healthcare), respectively. In all samples, 4′,6′-diamino-2-phenylindole hydrochloride was added at 100 ng ml^−1^ to visualise DNA in the cell nucleus. Images were obtained using a fluorescence microscopy Axiovert 200 (Zeiss, Jena, Germany) and a digital camera. MDCK cells transfected with a vector containing the polyserase-1 cDNA (Cal *et al*, 2003) were used as negative control.

### Scrape-wound migration assay and matrigel invasion assay

Monolayers of MDCK/DESC1 cells or control cells were scraped with a pipette tip in a single stripe. The wounded cultures were incubated at 37°C and cells migrating into the scraped area were imaged at different times. Tumour cell invasion assay was performed in cell culture chambers coated with matrigel (BD Biosciences, San Jose, CA, USA) following manufacturer instructions. In brief, MDCK/DESC1 transfected cells and control cells were seeded (5 × 10^4^ cells well^−1^) in DMEM medium containing 5% BSA and 50 ng ml^−1^ HGF. A 10% FBS was additionally included in the lower chambers to act as a chemoattractant. After 48 h of incubation, the cells on the lower membrane surface were fixed and stained with 0.5% crystal violet in 10% methanol. Randomly selected microscopic fields (magnification × 20) were imaged to calculate the average number of occupied pores per microscopic field. Data are expressed as mean±s.e.m. Differences in value distribution were statistically validated using the two-tailed *t*-test for unpaired data. Statistical analyses were performed using the program GraphPad Prism 3 for Windows. Difference was considered to be significant at values lower than *P*<0.05.

### Analysis of morphological changes in a 3D collagen lattice

MDCK/DESC1 cells or control cells were assayed to evaluate their ability to form tubular structures within a 3D Collagen Cell Culture System (Chemicon International, Temecula, CA, USA), following manufacturer instructions. Briefly, 1 × 10^3^ cells were mixed with a neutralised chilled collagen solution, dispensed into 96-well plates and incubated at 37°C until gelification. Culture medium containing 3% FBS and HGF (30 ng ml^−1^) or without HGF (control) was added to the wells and renewed every day for 7 days. Cultures were photographed after 7 days under phase contrast using an inverted microscope. To semiquantify the formation of tubular structures, 15 microscopic fields were randomly selected per experimental condition. Then, the number of tube structures was determined, and their length was also measured by using image analysis software.

### Immunohistochemistry

Human tissues were obtained as formalin-fixed, paraffin-embedded tissue sections from the archives of the tumour bank from the Hospital Universitario Central de Asturias (HUCA, Oviedo, Asturias, Spain). Tissue arrays were likewise used to evaluate DESC1 expression according to the histological type of cancer. After dewaxing and rehydrating, samples were blocked in 15% goat serum and then incubated overnight with anti-DESC1 antibody (1 : 150). Then, sections were incubated with an EnVision System labelled polymer-HRP anti-rabbit (DAKO, Glostrup, Denmark), and the staining was completed by incubation with diaminobenzidine colorimetric reagent (DAKO), followed by counterstaining with hematoxylin. Finally, the slides were dehydrated and mounted. Controls included samples that were incubated with a preimmune serum.

## RESULTS

### Production of the recombinant DESC1 catalytic domain and generation of polyclonal antibodies

**Figure 1:**
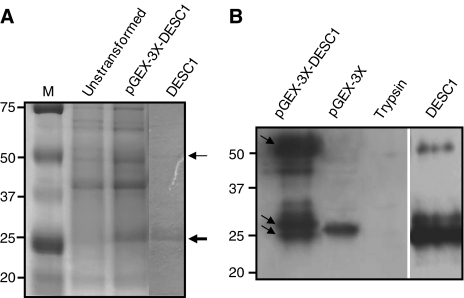
Production of recombinant DESC1. (**A**) Untransformed BL21(DE3)pLysE *E. coli* cells (lane 2) and cells transformed with pGEX-3X-*DESC1* after IPTG induction (lane 3) or purified DESC1 (lane 4) were analysed by SDS–PAGE. The sizes of molecular weight marker (kDa) are indicated on the left (Lane 1, M). DESC1 fused to GST is indicated with a thin arrow. Position for DESC1 released from GST is indicated with a thick arrow. (**B**) Western blot analysis of the proteins using the anti-DESC1 antibodies generated in this work. Fused GST+DESC1 protein (50.4 kDa) and released GST (26 kDa) and DESC1 (25.4 kDa) are indicated with arrows (lane 1). The generated antibodies detect GST expressed alone (lane 2), but not trypsin (lane 3). Lane 4, purified products eluted from a glutathione-Sepharose 4B column.

Molecular cloning of the human *DESC1* full-length cDNA was carried out by PCR amplification using EST BG697702 as template. The amplified product was 1269-bp long and contained the open reading frame reported previously (Lang and Schuller, 2001). The catalytic domain of this protein was expressed independently from the rest of the molecule following a strategy previously used to analyse other members of this family of proteases (Velasco *et al*, 2002). To this end, a 695-bp fragment coding for this region was PCR amplified and subcloned into the pGEX-3X vector generating the plasmid pGEX-3X-*DESC1*, which allows the expression of this catalytic domain fused to the glutathione-*S*-transferase. This region contains 231 amino acids (25.4 KDa), starting at the Ile191 after the putative proteolytic activation site (Arg190) within the conserved R-IVGG motif of this type of serine proteases (Netzel-Arnett *et al*, 2003). A 51.4 kDa band corresponding to the combined molecular masses of GST (26 kDa) and DESC1 (25.4 kDa) was detected by SDS–PAGE (Figure 1A). This fusion protein was purified by affinity chromatography on a glutathione-Sepharose 4B column (Figure 1A). DESC1 was not released from the GST with Factor Xa to avoid any minor contamination of this protease in the subsequent analysis of DESC1 activity. However, DESC1 was spontaneously released from the GST probably as a consequence of an autocatalytic processing during the course of the different assays (Figure 1A). This effect has been described for other TTSPs such as matriptase (Takeuchi *et al*, 1999), matriptase-2 (Velasco *et al*, 2002), matriptase-3 (Szabo *et al*, 2005), TMPRSS2 (Afar *et al*, 2001) and TMPRSS3 (Guipponi *et al*, 2002).

The DESC1 protein fused to GST was likewise used to generate rabbit polyclonal antibodies against human DESC1. The specificity of these antibodies was tested during the protein purification process by Western blot (Figure 1B). As expected from an autoactivation process, immunoreactive bands of 51.4, 26 and 25.4 kDa were clearly visible, corresponding to the fusion protein (GST+DESC1), and the released GST and DESC1, respectively. A 0.5 *μ*g of trypsin was added to the gel to be used as negative control. The produced antibodies also recognise the mix of GST and DESC1 after purification by affinity chromatography. This result reveals the ability of the generated antibody to recognise DESC1.

### DESC1 is a catalytically active serine protease that cleaves protein substrates

**Figure 2:**
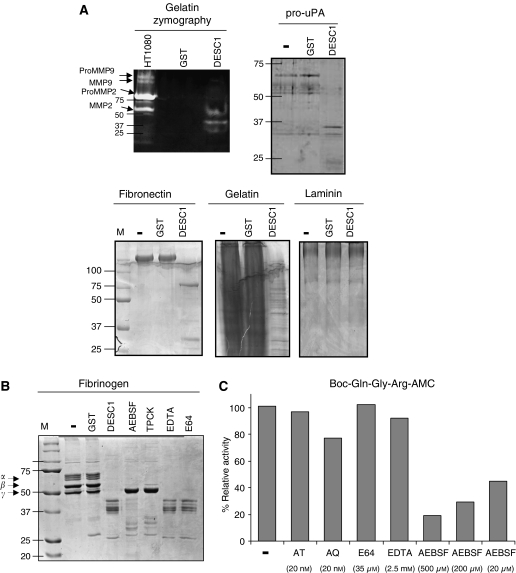
Analysis of enzymatic properties of DESC1. (**A**) Gelatin zymography: purified DESC1 has gelatinolytic activity. Purified GST was used as negative control and 5 *μ*l of the conditioned medium of the cell line HT1080 was used as positive control. ProMMP9 and MMP9, and ProMMP2 and MMP2 gelatinolytic activities are indicated with an arrow. Fibronectin, type I gelatin, laminin and pro-uPA were incubated alone (−) or in presence of purified GST or DESC1. Molecular weight markers (M, kDa) are shown on the left and for pro-uPA, on the right. (**B**) Fibrinogen was also incubated alone (−) or in presence of GST (lane 2) or DESC1 (lane 3). Preincubation for 30 min at 37°C with 100 *μ*M AEBSF (lane 4), 0.2 mM TPCK (lane 5), 2.5 mM EDTA (lane 6) or 10 *μ*M E64 (lane 7) was carried out before incubation with the substrate. GST and DESC1 positions are indicated on the right. (**C**) Inhibition assay using the synthetic fluorescent peptide Boc-Gln-Gly-Arg-AMC as susbstrate. Recombinant DESC1 was preincubated in the presence or absence of the protease inhibitors as indicated in Material and Methods. (−) indicates absence of inhibitor.

Enzymatic activity of DESC1 was demonstrated through different assays. Thus, a gelatine zymogram indicated that this serine protease displayed a prominent gelatinolytic activity. By contrast, the GST alone, expressed and purified in parallel to be used as a negative control, did not show any activity (Figure 2A). Then, DESC1 recombinant protein was incubated with different protein substrates, including extracellular matrix protein components, and the products of the reactions were visualised by SDS–PAGE gels. As Figure 2A shows, DESC1 hydrolyses fibronectin, gelatin, pro-UPA, but not laminin. The enzyme may likewise hydrolyse fibrinogen (Figure 2B). However, proteolytic activity on these substrates was not detected when incubated with GST alone (Figure 2A and B). These results suggest a potential role for DESC1 in the degradation and/or remodelling of the extracellular matrix ECM. Furthermore, the cleavage products obtained from DESC1 incubation with the single-chain pro-uPA (Figure 2A) resembled the size of the two chain forms (20 and 33 kDa) of the enzymatically active uPA capable of interacting with the uPA receptor, a system which is known to play a critical role in cancer progression. Preincubation of DESC1 with serine proteases inhibitors such as AEBSF or TPCK produces a considerable reduction in the *γ*-chain of fibrinogen cleavage (Figure 2B). By contrast, this effect was not observed when the preincubation was carried out with inhibitors for other types of proteases like EDTA and E64 (Figure 2B).

We have also examined the ability of DESC1 to degrade peptide substrates (Figure 2C). The predicted preferential cleavage for other TTSPs at amino acid residues with positively charged side chains was tested for DESC1 using two synthetic AMC-coupled peptides with Arg residues as P1 site. The results indicated that DESC1 was able to cleave Boc-Gln-Gly-Arg-AMC more efficiently than Boc-Gln-Ala-Arg-AMC (data not shown), with a *K*_m_ of 134 *μ*M and *V*_max_ of 3 × 10^−4^ *μ*M s^−1^. The proteolytic activity of DESC1 using the first peptide substrate was inhibited by preincubation with the serine proteinase inhibitor AEBSF (above 80% inhibition at 500 *μ*M). These results confirm the serine protease nature of DESC1.

### Membrane localisation and effect of DESC1 expression on motility of MDCK cells

**Figure 3:**
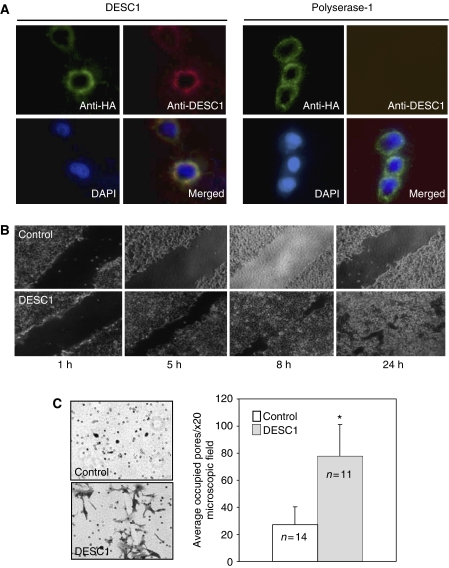
Membrane localisation and effect of DESC1 expression on MDCK cells motility. (**A**) Immunocytochemical detection of recombinant DESC1 expression in MDCK cells. The images were captured by fluorescence microscopy of MDCK cells transfected with pcDNA-HA-*DESC1* vector or with the same pcDNA3-HA plasmid containing the cDNA for polyserase-1. Immunofluorescent detection of anti-HA antibodies was carried out with a fluorescein-conjugated anti-mouse antibody, and detection of anti-DESC1 antibodies with a Texas Red-conjugated anti-rabbit antibody. Result shows the membrane localisation of DESC1. (**B**) Wound closure assay. Scrape wounds were made in confluent monolayers of cells stably transfected with vector containing *DESC1* or control vector. Cell layers were imaged at the indicated times. (**C**) Matrigel invasion assay. Invasion capacity of MDCK/DESC1 cells and control transfectants were analysed by a Matrigel invasion assay after 48 h of incubation. Number of occluded pores by the cells and by the control cells transfected were counted and represented on the right (^*^P<0.0001). *N* indicates the number of microscopic fields analysed.

MDCK cells were stably transfected with DESC1 cDNA (MDCK/DESC1 cells) to examine the possible contribution of this protease to cellular invasiveness. Expression of exogenous DESC1 was confirmed by immunostaining using the generated anti-DESC1 antibody and an anti-HA antibody (Figure 3A). MDCK cells transfected with a vector containing the cDNA encoding for polyserase-1 was used as control. In all cases, the samples were not permeabilised to detect the presumably extracellular catalytic domain. Two positive clones were selected for further analysis and results were equivalent in both cases. These assays were carried out using two different approaches. First, these clones were grown to confluence to carry out a scrape-wound migration assay. After *in vitro* ‘wounding’ of the cell monolayers, the cultures allowed to grow and wound closures were visualised at different times. As can be seen in Figure 3B, MDCK/DESC1 migrated to nearly cover the wound site within 8 h. By contrast, wound closure was incomplete after the same time interval in control cells (MDCK cells stably transfected with an empty vector), remaining almost intact after 24 h. These data suggest that DESC1 may be involved in migration and motility properties of these cells.

The second approach consisted in analysing cell behaviour in a matrigel invasion assay. In this case, we used invasion chambers to evaluate the ability of MDCK/DESC1 cells to degrade matrigel. After 48 h of incubation and 10% FBS used as chemoattractant, the migratory cells were stained and the number of pores occupied in randomly selected 20 × microscopic fields were counted and averaged (Figure 3C). A significant difference was observed between the levels of migration of the MDCK/DESC1 when compared to the MDCK cells transfected with the empty vector. Likewise, MDCK/DESC1 cells not only could extravasate the matrix but they also showed a branched and more elongated morphology than control cells. This tendency was also apparent at 24 h of incubation and also when lowering the chemoattractant concentration to 2% (not shown).

### MDCK cells expressing DESC1 form cysts that display long extensions in a 3D collagen lattice

**Figure 4:**
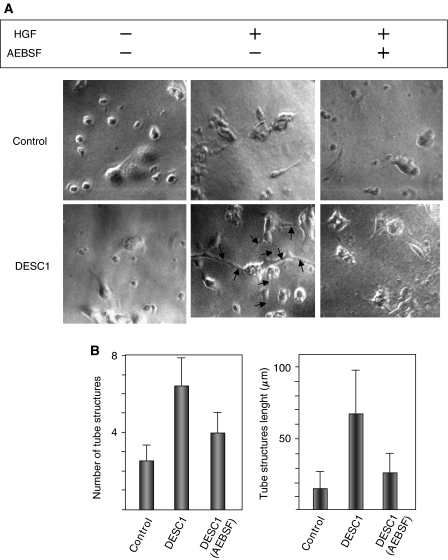
(**A**) HGF-stimulated MDCK/DESC1 cells embebbed in collagen gel form tubular structures. MDCK cell clones stably transfected with control vector or expressing DESC1 were cultured in a type I collagen matrix for 7 days in presence (+) or in absence (−) of 30 ng ml^−1^ of HGF, and in the presence (+) or absence (−) of 50 *μ*M of AEBSF. The branching extensions formed by MDCK/DESC1 cells are indicated by arrows. (**B**) Quantification of the number and length of tubular structures of 15 randomly selected microscopic fields following HGF stimulation.

MDCK cells have been reported to form cysts and develop branching tubules following HGF stimulation when they are grown embedded in collagen gel network (Ridley *et al*, 1995). We wanted to evaluate the ability of DESC1 to induce these effects in MDCK cells. To test this, MDCK/DESC1 or control cells were mixed with a collagen gel and allowed to grow. These assays were carried out with or without HGF treatment and the cultured cells were imaged after 7 days. As can be seen in Figure 4A, MDCK/DESC1 cells form cysts from which surface-long branching extensions are clearly detectable following HGF stimulation. In terms of number of tube structures, DESC1 expression induces the formation of more tubes than control cells (average of 6.5 *vs* 3.5, respectively). However, the difference is considerably higher in terms of tube lengths (60.8 *μ*m *vs* 10.5 *μ*m). Moreover, the formation of these extensions clearly diminished with the presence 50 *μ*M of the serine proteinase inhibitor AEBSF (Figure 4A and B). These data would indicate that DESC1 expression enhances the capacity of MDCK to invade a collagen matrix following HGF stimulation.

### Analysis of DESC1 expression in normal and tumoral human tissues

**Figure 5:**
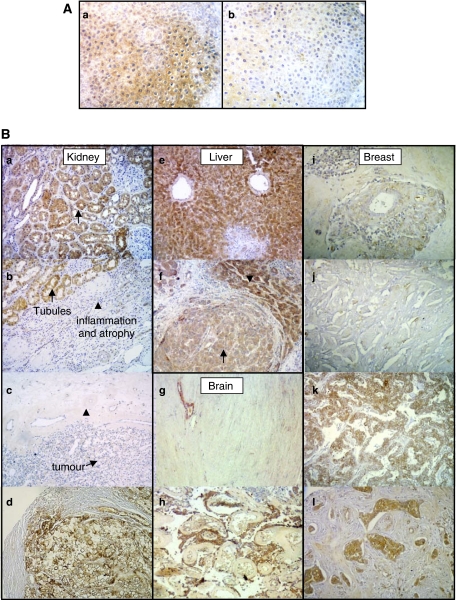
DESC1 expression in kidney, liver, brain and breast normal and tumour tissues. (**A**) (a) Immunostaining of the epithelial component of a larynx tissue sample using the antibody generated in this work. (b) Immunostaining performed with a preimmune control serum does not show this pattern. (**B**) (a–d) IHC in kidney. (a) Normal kidney showing DESC1 expression in tubules (arrow). (b) Reactive tubules show positive expression (arrow), the proximal inflammated and atrophical region is negative (arrowhead in b and c), and the derived well-differentiated clear cell tumour in (c) is negative (see arrow). (d) Sample from a tissue array slide showing positivity in a poorly differentiated renal clear cell tumour. Note the negativity in the stroma. (e–f) IHC in liver. (e) Liver tissue showing intense signal in the pericentral hepatocytes. (f) Renal invasive hepatocarcinoma corresponding to the same specimen as in (e). Staining diminishes in the nodal satellite (arrow) that compresses some positive normal hepatocytes (arrowhead). (g–h) IHC of the brain. (g) Leptomeninges (dura mater) showing negative expression of DESC1. (h) Atypical meningioma showing positivity. (i–l) IHC of breast. (i) Reactive benign breast tissue showing slight positivity surrounding a negative duct. (j) Negative immunostaining in a ductal micropapilar breast tumour. (k) Positivity in a ductal breast carcinoma. (l) Positivity in a lobular breast carcinoma. Magnification was 20 × and counterstaining was with haematoxylin.

DESC1 expression in oropharyngeal cavity correlates directly with keratinocyte differentiation and inversely with squamous cell carcinoma progression (Sedghizadeh *et al*, 2006). However, our data suggest that DESC1 could be implicated in the development and/or progression of some type of tumours. These facts prompted us to extend the study of DESC1 expression to different type of tissues including kidney, liver, brain and breast. This study was carried out using the antibodies generated in this work and, to determine optimal antibody dilutions, we first employed a normal larynx tissue sample that showed an immunostaining of the epithelial component (Figure 5A). This signal was absent when the sample was treated with preimmune serum (Figure 5A). Then, we employed paraffin-embedded tissue and tissue arrays that included specimens of normal and squamous carcinoma, adenocarcinomas and other tumours of different histological type. Figure 5Ba–d shows DESC1 expression in normal and tumoral kidney. Normal tubule epithelial cells showed intense expression (Figure 5Ba; arrow). Inflammation and atrophy represented in Figure 5Bb and c (arrowhead) correlate with a dramatic loss of expression that is also apparent in the derived clear cell tumour (Figure 5Bc, arrow). We tested two negative renal tumours versus six positive cases (see example in Figure 5Bd). In the case of the liver, Figure 5Be reveals intense staining in normal hepatocytes. A nodule from an invasive hepatocarcinoma displays a clear diminished expression (arrow in Figure 5Bf). More intense immunoreactivity can be seen in normal hepatocytes compressed by the tumour nodule (arrowhead). In brain tissue, expression was negative for the normal tissue (Figure 5Bg) but positive in this case for an atypical meningioma (Figure 5Bh). Finally, in normal breast tissue, DESC1 expression was restricted to glands and duct epithelium to a lesser extent. Figure 5Bi represents a reactive breast tissue with a very weak expression surrounding a negative duct. With respect to tumours, a varied panorama of negative (Figure 5Bj) and positive (Figures 5Bk and l) DESC1 expression was observed (7 negative *vs* 12 positive cases). Moreover, expression in breast depended on the tumour histological type, being mainly restricted to ductal and lobular variants, but other additional factors may also contribute as not all of the ductal tumours analysed were positive. Additionally, two cases of ductal carcinoma *in situ* showed increased expression with respect to the corresponding infiltrating tumour. Data obtained from this immunohistochemistry analysis suggest that both up- and downregulation of DESC1 could be associated to cancer progression.

## DISCUSSION

Proteolytic activities have been traditionally associated with the growth and expansion of different type of tumours. However, different serine proteases have been found downregulated in different types of human carcinomas. This is the case of DESC1, the expression of which inversely correlates with progression of head and neck tumours (Sedghizadeh *et al*, 2006). Our interest in this type of neoplasias prompted us to investigate the implication of DESC1 in cancer biology through the study of its activity, effect on invasiveness of a known cell model and the analysis of its expression in a variety of tumour tissues.

Common with most of the TTPS (Szabo *et al*, 2003) endogenous targets for human DESC1 remain unresolved. However, the production and purification of the recombinant catalytic domain of this protease has revealed for the first time that it exhibits a significant *in vitro* activity for substrates such as fibronectin, gelatin or fibrinogen. The ability to degrade extracellular matrix components has been observed in members of the TTSP family such as matriptase (Lee *et al*, 2000) or matriptase-2 (Velasco *et al*, 2002). This suggests that DESC1 could participate in the degradation of the extracellular matrix that occurs in normal and pathological conditions, including cancer. Likewise, DESC1 also shares with these two enzymes the capability for pro-uPA activation, revealing the possibility that DESC1 could participate in proteolytic cascades mediated by activated uPA.

Our initial results prompted us to use the full-length DESC1 in cell-based assays due to the fact that activity of the catalytic domain expressed in a bacterial host does not accurately reflect the enzyme activity *in vivo*. Moreover, the use of these types of assays does not remove the possible influence of the ancillary domains on the substrate specificity. We chose the dog kidney epithelial cell line MDCK, which have been widely used to attempt to elucidate the functional role in tumour processes of different proteins, including membrane-associated metalloproteases (Kadono *et al*, 1998; Kang *et al*, 2000). After the selection of two clones that expressed exogenous DESC1, we carried out three different types of assays. First, we observed an increase in the migration and motility properties of the clones that produce the protease, in a scrape-wound migration assay respecting to the control cells. Then, in matrigel-based invasion assays we found that MDCK/DESC1 transfectants acquired an enhanced capacity to migrate and extravasate the collagen matrix, adopting a branched and elongated morphology. In a third type of assay, we showed of these cells to form branching extension in a 3D collagen lattice. The formation of this type of extensions represents a partial epithelial–mesenchymal transition, which could finally induce tubulogenesis (O’Brien *et al*, 2004). This is a phenotype relevant to renal development and carcinogenesis, and takes place concurrent with complex morphogenic processes that require cell proliferation and movement, rearrangements of the cytoskeleton and of the cell–cell junctional complexes, and remodelling of the cell matrix (Birchmeier *et al*, 2003). Results obtained in these assays evidence for the first time that DESC1 is a serine protease that could be involved in motility, cell growth and invasion processes.

Since DESC1 was identified as a gene downregulated in carcinomas of the head and neck, and the data obtained in this work indicate a possible involvement of this protease in the tumorigenic process, we carried out an immunohistochemical analysis of DESC1 expression in a variety of tissues of different origin. Although we could confirm this downregulation in larynx and pharynx tissues (not shown), the study revealed for the first time overexpression of DESC1 in a variety of carcinomas like kidney, brain and breast cancer. The up/downregulation for a particular protease among different types of cancers has been reported for other serine proteases, including some TTSPs. Thus, loss of testisin expression has been found in testicular tumorigenesis (Hooper *et al*, 1999), but its expression is associated with advanced stages of ovarian cancer (Shigemasa *et al*, 2000). Matriptase is upregulated in prostate and cervical cancer (Santin *et al*, 2003), but it is downregulated in advanced-stage ovarian tumours (Tanimoto *et al*, 2005). Hepsin has been described to promote cancer progression and metastasis in a mouse model (Klezovitch *et al*, 2004), but it seems to inhibit cell growth and invasion in prostate cancer cells (Srikantan *et al*, 2002). Moreover, we can not rule out that DESC1 could likewise be involved in proteolytic cascades that would include protease inhibitor as happens with matriptase (Netzel-Arnett *et al*, 2006). In this regard, the mouse counterpart of this protease, mDESC1, forms stable inhibitory complexes with both plasminogen activator inhibitor-1 and protein C inhibitor (Hobson *et al*, 2004). These observations raise new intriguing questions about the role of DESC1 in normal and pathological processes. To finally corroborate these data, further exhaustive functional studies and analysis of its expression in more tissues, including different histopathological parameters, would be needed to assess the final balance of DESC1 expression in each type of tissue. Moreover, we have shown in this study that the generated antibodies do not recognise the three serine protease domains of polyserase-1. However, it cannot be ruled out that these antibodies could recognise other TTPS, specially the closely related members of the HAT/DESC subfamily. Additionally, the generation of the mouse lacking this enzyme could contribute to shed light on the up- and downregulation of DESC1 in different tumours, and about its possible involvement in complex proteolytic cascades.
